# Uptake of **^18^**FDG by a Hepatic Adenoma on Positron Emission Tomography

**DOI:** 10.1155/2011/276402

**Published:** 2011-12-22

**Authors:** J. A. Stephenson, T. Kapasi, O. Al-Taan, A. R. Dennison

**Affiliations:** ^1^Department of Radiology, Leicester General Hospital, University Hospitals of Leicester, Leicester LE5 4PW, UK; ^2^Department of Hepatobiliary Surgery, Leicester General Hospital, University Hospitals of Leicester, Leicester LE5 4PW, UK

## Abstract

Fluorodeoxyglucose positron emission tomography (^18^FDG-PET) is currently the best noninvasive test to confirm hepatic metastases when diagnostic uncertainty exists after initial imaging with first-line modalities. However, we report the second case of “false-positive” uptake of ^18^FDG on PET scanning from a histopathologically confirmed hepatic adenoma.

## 1. Introduction

There are many reports, which have demonstrated the value of using fluorine-18 (F18) fluorodeoxyglucose positron emission tomography (^18^FDG-PET) for the assessment of metastatic disease in patients with Hürthle cell carcinoma of the thyroid gland (HCT). FDG-PET is currently the best noninvasive test to confirm hepatic metastases when diagnostic uncertainty is present. However, we report the first of “false-positive” uptake of ^18^FDG on PET scanning in a patient the HCT and concurrent liver lesions presumed metastatic in origin.

## 2. Case Report

A 34-year-old female with no other medical comorbidities presented with an enlarged multinodular unilateral thyroid goitre. Fine-needle aspiration (FNA) biopsy was used to assess this enlargement, which demonstrated a follicular neoplasm (THY3) on cytology. A diagnostic lobectomy for formal histopathological diagnosis established features of a widely invasive follicular HCT. In view of the findings of malignancy, a completion thyroidectomy was performed, which did not exhibit any evidence of contralateral disease. Treatment was completed by adjuvant ^131^I radio-ablative therapy.

**Figure 1:**
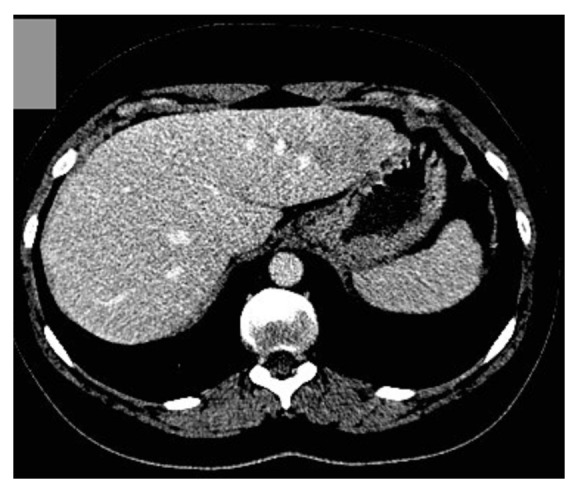
CT scan showing hypovascular lesion in left lobe of liver.

**Figure 2:**
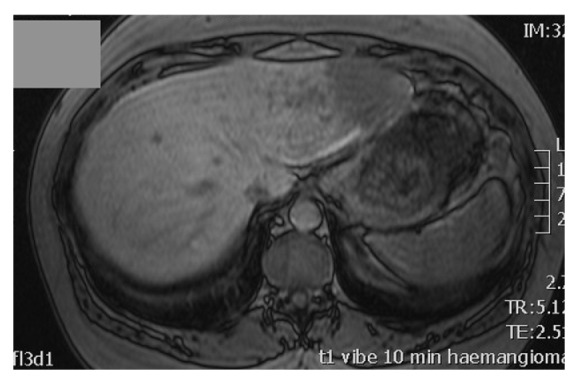
MRI scan showing abnormal areas in segments two, three, and four of the liver.

**Figure 3:**
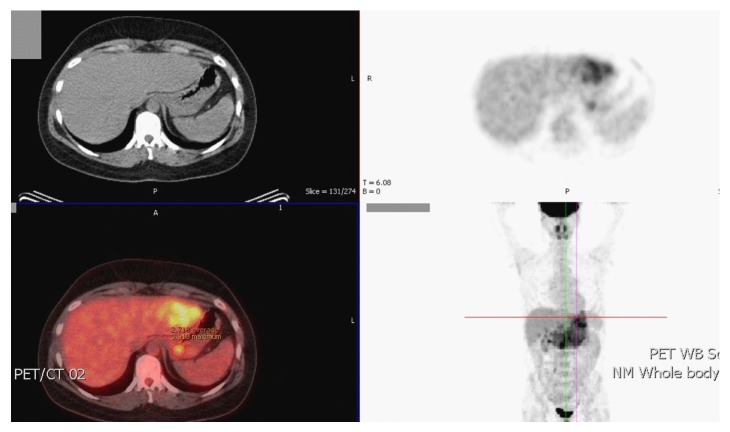
PET CT showing areas of increased ^18^FDG uptake correlating to lesions in segments two, three, and 4 of the liver.

A staging arterial phase computed tomography (CT) scan at this time demonstrated enlargement of the left lobe of the liver, which contained at least three hypovascular masses measuring between 2 and 3 cm in diameter (Figure 1). Repeated imaging of the liver found no new abnormality, and a magnetic resonance imaging (MRI) scan was obtained to further characterise the nature of this heterogeneous attenuation pattern. MRI demonstrated abnormal areas in segments two, three, and four of the liver and suggested the appearances are most likely to be those of metastatic disease (Figure 2). A whole-body ^131^Iscintigraphy scan did not show any uptake into the liver. Furthermore, the patients' thyroglobulin level was below one. A percutaneous ultrasound- (US-) guided biopsy of the liver lesions did not demonstrate any evidence of malignancy. Since cross-sectional imaging was strongly suspicious of metastatic disease, an FDG-PET scan was performed to assess for the presence of highly metabolic areas that would in most cases be suggestive of malignancy. On PET (Figure 3), the lesions identified on cross-sectional imaging were found to demonstrate an SUV_max_ of 3.9, thus presumed malignant in origin.

In view of the conflicting evidence as to whether these lesions were metastatic deposits or not, the patient underwent a laparoscopy, intra-operative ultrasound (IOUS), and biopsy of the liver lesions to gain a definitive tissue diagnosis, prior to making a decision on whether to proceed to formal liver resection accepting the associated morbidity and mortality of this procedure. Laparoscopy and IOUS assessment reviled no extra hepatic disease and confirmed the presence of the 3 masses within segments 2, 3, and 4 of the liver. Multiple IOUS-guided core biopsies were taken from each lesion for histological analysis. All sections of tissue were proven to be hepatic adenomas and *not* metastatic deposits.

The patient made an uneventful recovery from the laparoscopy. The patient underwent a CT at six months post laparoscopy and these lesions showed no change in size, shape, or characteristics and there were no new lesions. Interval US scanning will be performed to monitor the lesions.

## 3. Discussion

Hürthle cell carcinoma of the thyroid gland (HCT) is a subtype of follicular thyroid carcinoma. Like all lesions-graded THY3 (follicular) on cytology, they cannot be assessed as being malignant or benign without pathological assessment. The tumour consists of mainly oxyphilic follicular cells [2] and is more likely to exhibit metastatic tendencies when compared with other differentiated thyroid tumours [3–6]—in one report the incidence of metastatic disease in the HCT variety was found to be 33% [7]. However, the behaviour amongst the malignant HCT subtype is difficult to predict, and no clinical or pathological feature can predict behaviour [8]. A total thyroidectomy for malignant HCT, as in this patient, is the accepted form of surgical treatment [3, 9]. Adjuvant radio-ablative treatment with iodine is also known to improve outcomes in such patients [10].

Hürthle cells produce thyroglobulin (Tg). Tg is a sensitive (91%) and specific (99%) marker when used as a marker of recurrence in patients with previous or recurrent differentiated thyroid carcinoma [11]. Tg levels should be undetectable in patients who have had a thyroidectomy and ^131^I ablation but low levels, as in this patient (Tg 1 ng/mL), may signify the presence of residual normal thyroid tissue rather than residual or recurrent disease. It is perhaps the trend in Tg measurements postoperatively that is more useful to detect recurrence/residual disease rather than a single isolated low Tg assay.

Iodine-131 whole-body scintigraphy (WBS) is an established method of detecting metastases and/or recurrence from differentiated thyroid carcinoma [12] with high specificities [13]. However, a negative WBS as seen in this patient cannot exclude the presence of metastatic disease, as this modality is only moderately sensitive for iodine-positive metastatic disease with quoted sensitivity if executed well in the region of 50% [13, 14].

CT can characterise between malignant and benign hepatic lesions. Hepatic adenomas are usually iso-attenuating lesions—except in the presence of fat when they may appear to be hypo-attenuating [15]. In this patient's case, further characterisation would be required through further cross sectional imaging.

There are many reports, which have demonstrated the value of using FDG-PET studies for the assessment of metastatic disease in patients with HCT, particularly when any potential tumour has low iodine avidity [16–20]. The technique is currently the best noninvasive test to detect hepatic metastases from gastrointestinal malignancy [21]. One meta-analysis, which included the results from a multicentre study (for the detection of recurrent HTC by FDG-PET) demonstrated a sensitivity of 92% and a specificity of 80% [18]. One investigator suggests that *all* patients should undergo PET as part of postoperative staging and in long-term followup [19]—these benefits are amplified in those patients who exhibit elevated Tg assays. The intense uptake of ^18^FDG suggests the presence of either recurrent of metastatic disease and this is indicative of a poor prognosis [19]. Now the importance of FDG-PET in assessing for recurrence and/or metastasis in HTC has been established, it is possible to understand the importance and implications of a false-positive result, as in this case report. There exists only one confirmed case in the literature of false-positive uptake of FDG in a hepatic adenoma, which was thought to be a metastatic deposit from previous breast carcinoma until surgical biopsy confirmed the benign nature of the lesion [22].

This false-positive result highlights the importance of laparoscopic biopsy in determining the nature of a liver lesion. The benefits of laparoscopy and IOUS, such as optimizing patient selection for hepatectomy of curative intent [23], are well recognised and beyond the context of this discussion.
